# Supplementary material for: Human behaviour, NPI and mobility reduction effects on COVID-19 transmission in different countries of the world
Source: BMC Public Health. 2022 Aug 22;22:1594. doi: 10.1186/s12889-022-13921-3 (PMC9394048; doi:10.1186/s12889-022-13921-3)
Supplement: Supplementary file 1 — Additional file 1. Appendix [file 12889_2022_13921_MOESM1_ESM.pdf]

,

1 Appendix

1.1 Next generation matrix and reduced Jacobian

The Jacobian matrix near the disease-free equilibrium (DFE, which consists of $S(0) = N$ and $I(0) = 0$) for the system of equations (3) is:

$$J = \begin{pmatrix} 0 & 0 & -\beta & 0 & 0 \\ 0 & -\sigma & \beta & 0 & 0 \\ 0 & \sigma & -\gamma + \epsilon(\gamma - \kappa)(1-a) & 0 & 0 \\ 0 & 0 & \epsilon\kappa(1-a) & -\gamma & 0 \\ 0 & 0 & \gamma(1-\epsilon(1-a)) & \gamma & 0 \end{pmatrix}$$

Using the next generation matrix method around the DFE, ([1]) we compute R_0 as the largest eigenvalue of the matrix FV^{-1}. F and V are known as the transmission and the transition part, respectively. Consider a disease free population and an infected individual enter into a compartment, then each entry of matrix F represents the production of new infections between compartments, and each entry of matrix V^{-1} describes changes of this individual in each compartment. Then each entry of matrix FV^{-1} describes the expected number of infections that produced by the original entry infected. [2].

we obtain the largest eigenvalue of the matrix FV^{-1}, closed form expression:

$$R_0 = \frac{\beta}{(\epsilon\gamma a - \epsilon\kappa a - \epsilon\gamma + \epsilon\kappa + \gamma)}. \tag{1}$$

Further, using [3], we can compute the eigenvalues of the reduced Jacobian above and find that there is one positive eigenvalue (responsible for the growth near the DFE) which can be derived in closed form:

$$\rho = \frac{-\epsilon\gamma a + \epsilon\kappa a + \epsilon\gamma - \epsilon\kappa - \gamma - \sigma}{2} +$$

$$\sqrt{\frac{\epsilon^2\gamma^2 a^2 - 2\epsilon^2\gamma\kappa a^2 + \epsilon^2\kappa^2 a^2 - 2\epsilon^2\gamma^2 a + 4\epsilon^2\gamma\kappa a - 2\epsilon^2\kappa^2 a + \epsilon^2\gamma^2 - 2\epsilon^2\gamma\kappa + \epsilon^2\kappa^2}{4}}$$

$$+\sqrt{\frac{2\epsilon\gamma^2 a - 2\epsilon\gamma\kappa a - 2\epsilon\gamma a\sigma + 2\epsilon\kappa a\sigma - 2\epsilon\gamma^2 + 2\epsilon\gamma\kappa + 2\epsilon\gamma\sigma - 2\epsilon\kappa\sigma + 4\beta\sigma + \gamma^2 - 2\gamma\sigma + \sigma^2}{4}},$$

which in turn can be solved for an expression of β as a function of the growth factor ρ near the DFE:

$$\beta = \beta(\rho) := \frac{\epsilon\gamma a\rho + \epsilon\gamma a\sigma - \epsilon\kappa a\rho - \epsilon\kappa a\sigma - \epsilon\gamma\rho - \epsilon\gamma\sigma + \epsilon\kappa\rho + \epsilon\kappa\sigma + \gamma\rho + \gamma\sigma + \rho^2 + \rho\sigma}{\sigma}.$$

Finally we can estimate R_0 as a function of the growth factor near the DFE in each region using (1) as:

$$R_0(\rho) = \frac{\epsilon\gamma a\rho + \epsilon\gamma a\sigma - \epsilon\kappa a\rho - \epsilon\kappa a\sigma - \epsilon\gamma\rho - \epsilon\gamma\sigma + \epsilon\kappa\rho + \epsilon\kappa\sigma + \gamma\rho + \gamma\sigma + \rho^2 + \rho\sigma}{\sigma((\epsilon\gamma a - \epsilon\kappa a - \epsilon\gamma + \epsilon\kappa + \gamma))}. \tag{2}$$

1.2 Mask Efficacy and Compliance Data

During the first several months of the pandemic there was considerable debate on the effect of face masks on limiting the spread of the COVID-19 pandemic. There was also debate on whether to recommend the general public to use a face mask. Later, articles, scientific reports, and data proved the impact of face masks in altering the outcomes of peak hospitalization [4]. There have been observational studies in health care workers which reported that wearing surgical masks and N95 masks can reduce the risks of respiratory illnesses by 40–60% [5].

Notably, face masks are found to be useful in both preventing asymptomatic transmission and illness in healthy persons. Moreover, varying efficacy and compliance of masks have an impact on the transmission dynamics and control of the COVID-19 pandemic [6]. A review [7] of observational studies estimates that surgical and comparable cloth masks are 67% effective in protecting the wearer. Some reports show that even a cotton T-shirt can block half of the inhaled aerosols and almost 80% of exhaled aerosols measuring $2\mu m$ across (e.g. unpublished work by Linsey Marr, an environmental engineer at Virginia Tech in Blacksburg). Furthermore, in [8] the mask wearing reduction factor is taken to be in a range of [30%, 80%], where 30% effectiveness is the level of a paper mask or 1-layer mask, while 80% and higher are surgical masks and N95 masks, which were not typically available to everyday individuals in 2020. We consider 50% to be the mask efficacy in our model .

The proportion of a population wearing face masks differs across countries/regions based on social norms, political reasons, the consequences of non-compliance e.g., fines. The results from a study surveying compliance are for example:

- The Institute for Health Metrics and Evaluation (IHME), a global health research center at the University of Washington [9] is reported the percentage of mask use in Italy was between 63% to 93% from September 1st till December 31, 2020, Sweden 1-7% , Saudi Arabia 73-76%, Ontario 75-85%, Florida 66-70%, Romania 63-86%, Ghana 50-36%, South Africa 80-81%, Indonesia 74-76%, Nepal 64-63%, Brazil 68-59%, Argentina 89-83%.
- According to data from the Institute for Health Metrics and Evaluation at the University of Washington in Seattle, mask use has held steady around 50% since late July in the United States. It was predicted to increase to 95% as of 23 September. (see [9]) Whereas, a survey from Gallup [10] shows 72% of U.S. adults say they either always wear a face mask or wear one often when going to public places.
- Percentage of people who worn a face mask outside their home always is reported 93.9% in Italy and 12.1% in Sweden 12.1% by YouGov; Imperial College London [11].

We adapt our SEIRL model with the compliance of mask-wearing value denoted as $compliance_m$ obtained from [9]. Table 1 represents the mask use for each region under study.

Author details

References

1. van den Driessche, P.: Reproduction numbers of infectious disease models. Infectious Disease Modelling **2**(3), 288–303 (2017)
2. Van den Driessche, P., Watmough, J.: Reproduction numbers and sub-threshold endemic equilibria for compartmental models of disease transmission. Mathematical biosciences **180**(1-2), 29–48 (2002)
3. Junling, M.: Estimating epidemic exponential growth rate and basic reproduction number. Infectious Disease Modelling (2020)
4. Eikenberry, S.E., Mancuso, M., Iboi, E., Phan, T., Eikenberry, K., Kuang, Y., Kostelich, E., Gumel, A.B.: To mask or not to mask: Modeling the potential for face mask use by the general public to curtail the covid-19 pandemic **5**, 293–308 (2020). doi:10.1016/j.idm.2020.04.001
5. Chan, K.H., Yuen, K.-Y.: COVID-19 epidemic: disentangling the re-emerging controversy about medical facemasks from an epidemiological perspective. International Journal of Epidemiology **49**(4), 1063–1066 (2020). doi:10.1093/ije/dyaa044. https://academic.oup.com/ije/article-pdf/49/4/1063/34275714/dyaa044.pdf
6. Stutt, R.O.J.H., Retkute, R., Bradley, M., Gilligan, C.A., Colvin, J.: A modelling framework to assess the likely effectiveness of facemasks in combination with ‘lock-down’ in managing the covid-19 pandemic **476**(2238) (2021). doi:10.1098/rspa.2020.0376
7. Chu, D.K., Akl, E.A., Duda, S., Solo, K., Yaacoub, S., Schünemann, H.J., et al.: Physical distancing, face masks, and eye protection to prevent person-to-person transmission of sars-cov-2 and covid-19: a systematic review and meta-analysis **395**(10242), 1973–1987 (2020). doi:10.1016/S0140-6736(20)31142-9
8. Wilson, A.M., Abney, S.E., King, M.-F., Weir, M.H., López-García, M., Sexton, J.D., Dancer, S.J., Proctor, J., Noakes, C.J., Reynolds, K.A.: Covid-19 and use of non-traditional masks: how do various materials compare in reducing the risk of infection for mask wearers? Journal of Hospital Infection **105**(4), 640–642 (2020)
9. The Institute for Health Metrics and Evaluation , Compliance with Mask. https://covid19.healthdata.org/ Accessed June 4, 2021
10. Brenan, M.: Americans' Face Mask Usage Varies Greatly by Demographics. https://news.gallup.com/poll/315590/americans-face-mask-usage-varies-greatly-demographics.aspx Accessed July 13, 2020
11. London, Y.I.C.: How Often Have You Worn a Face Mask Outside Your Home to Protect Yourself or Others from Coronavirus (COVID-19)? https://www.statista.com/statistics/1114375/wearing-a-face-mask-outside-in-european-countries/ Accessed January 10, 2021

Date	Ontario	Florida	Romania	Romania	Sweden	Italy	Ghana	SouthAfrica	SaudiArabia	Indonesia	Nepal	Brazil	Argentina
2/15/2020	0.01829	0.00000	0.00000	0.00000	0.00067	0.00000	0.00000	0.00000	0.00000	0.00000	0.09057	0.00000	0.00000
2/22/2020	0.02691	0.00000	0.00000	0.00000	0.00131	0.00001	0.00000	0.00000	0.00000	0.00000	0.11476	0.00000	0.00000
2/29/2020	0.03031	0.00003	0.00134	0.00134	0.00153	0.00419	0.00000	0.00000	0.00003	0.00000	0.12656	0.00014	0.00001
3/7/2020	0.03284	0.00357	0.06003	0.06003	0.00164	0.10944	0.00000	0.00047	0.00680	0.00000	0.13581	0.00549	0.00410
3/14/2020	0.03867	0.06427	0.21523	0.21523	0.00201	0.32249	0.00011	0.02506	0.14456	0.00000	0.15274	0.02726	0.13749
3/21/2020	0.05859	0.15733	0.32769	0.32769	0.00324	0.46549	0.01251	0.11553	0.34136	0.00126	0.20076	0.09729	0.35957
3/28/2020	0.09549	0.24071	0.42909	0.42909	0.00563	0.56049	0.10991	0.18993	0.46766	0.06344	0.28287	0.20706	0.47093
4/4/2020	0.13647	0.32407	0.52789	0.52789	0.00829	0.64554	0.20507	0.26154	0.52507	0.25560	0.37327	0.31499	0.56814
4/11/2020	0.17677	0.40636	0.62589	0.62589	0.01090	0.72940	0.28940	0.33213	0.54043	0.43113	0.46277	0.41410	0.66453
4/18/2020	0.21136	0.47686	0.70917	0.70917	0.01277	0.80137	0.36000	0.39356	0.57730	0.58007	0.54086	0.49739	0.74853
4/25/2020	0.22924	0.51036	0.72953	0.72953	0.01151	0.83583	0.38421	0.43554	0.56073	0.66219	0.58976	0.53799	0.80156
5/2/2020	0.24353	0.52466	0.69011	0.69011	0.00954	0.84639	0.38743	0.49456	0.51121	0.68499	0.61630	0.56103	0.83406
5/9/2020	0.26864	0.54407	0.69031	0.69031	0.00987	0.85674	0.40213	0.61091	0.51746	0.71006	0.61524	0.62100	0.85991
5/16/2020	0.28761	0.54414	0.73561	0.73561	0.01081	0.85564	0.44783	0.73377	0.54466	0.73027	0.60726	0.69211	0.87974
5/23/2020	0.29527	0.52847	0.77753	0.77753	0.01160	0.84670	0.50764	0.80117	0.55701	0.74153	0.61071	0.73016	0.88556
5/30/2020	0.31411	0.52907	0.79774	0.79774	0.01104	0.83249	0.51780	0.81907	0.58071	0.73926	0.61149	0.74696	0.88579
6/6/2020	0.33083	0.54140	0.78671	0.78671	0.01020	0.80731	0.50967	0.82133	0.67117	0.74383	0.62641	0.75529	0.88713
6/13/2020	0.34239	0.53483	0.76719	0.76719	0.01034	0.77781	0.51387	0.81890	0.76221	0.75501	0.62736	0.75559	0.88739
6/20/2020	0.35856	0.52207	0.75781	0.75781	0.01166	0.74461	0.53203	0.81319	0.79819	0.75467	0.60646	0.75303	0.89009
6/27/2020	0.37296	0.55413	0.70860	0.70860	0.01134	0.69003	0.55859	0.80587	0.80884	0.74341	0.56226	0.73893	0.89189
7/4/2020	0.39909	0.61166	0.63573	0.63573	0.01069	0.63430	0.55984	0.79760	0.79171	0.72624	0.52449	0.71849	0.88797
7/11/2020	0.46297	0.63923	0.62446	0.62446	0.00949	0.61156	0.56063	0.80223	0.77904	0.71961	0.53709	0.71143	0.88446
7/18/2020	0.55596	0.63657	0.64109	0.64109	0.00873	0.59740	0.56806	0.81170	0.77063	0.72150	0.54814	0.70721	0.88493
7/25/2020	0.63860	0.65527	0.65011	0.65011	0.00859	0.58686	0.57151	0.81479	0.76957	0.72326	0.54867	0.70213	0.88649
8/1/2020	0.68699	0.70307	0.63580	0.63580	0.00937	0.59280	0.56457	0.81241	0.77271	0.72517	0.55570	0.70147	0.88521
8/8/2020	0.71601	0.72661	0.62200	0.62200	0.00990	0.60069	0.55811	0.81006	0.76191	0.72414	0.58014	0.70231	0.88431
8/15/2020	0.72743	0.72371	0.62591	0.62591	0.01031	0.59081	0.54499	0.81436	0.74503	0.72297	0.61789	0.69583	0.88567
8/22/2020	0.72520	0.70189	0.63066	0.63066	0.00981	0.58864	0.51999	0.81403	0.73674	0.72424	0.64339	0.69074	0.88814
8/29/2020	0.73104	0.66906	0.63279	0.63279	0.00936	0.61310	0.50080	0.80359	0.72824	0.73144	0.64503	0.68797	0.88963
9/5/2020	0.74536	0.66257	0.63270	0.63270	0.01111	0.63671	0.50051	0.79614	0.73191	0.74014	0.63683	0.67933	0.89151
9/12/2020	0.75927	0.66843	0.63164	0.63164	0.01099	0.65226	0.49890	0.78153	0.72274	0.74837	0.63117	0.66529	0.89566
9/19/2020	0.76840	0.66789	0.63377	0.63377	0.00907	0.66943	0.46186	0.77644	0.72246	0.76343	0.64226	0.65516	0.89423
9/26/2020	0.76807	0.65840	0.63874	0.63874	0.00886	0.68881	0.44877	0.77859	0.72856	0.77424	0.63539	0.64643	0.89080
10/3/2020	0.78250	0.65061	0.64803	0.64803	0.00911	0.71563	0.46023	0.76847	0.71923	0.77107	0.61504	0.63600	0.88786
10/10/2020	0.80781	0.64671	0.65900	0.65900	0.00820	0.76616	0.45591	0.75711	0.71664	0.76113	0.61080	0.62469	0.88373
10/17/2020	0.80830	0.64379	0.67141	0.67141	0.00897	0.83617	0.43710	0.74680	0.71456	0.75020	0.62677	0.61071	0.88249
10/24/2020	0.80447	0.64354	0.70191	0.70191	0.01061	0.88397	0.42409	0.73191	0.72564	0.74649	0.64944	0.60226	0.88197
10/31/2020	0.81124	0.64409	0.75053	0.75053	0.01250	0.90621	0.40400	0.72804	0.74320	0.74183	0.64941	0.60144	0.87894
11/7/2020	0.82094	0.64823	0.78900	0.78900	0.01556	0.91790	0.37231	0.73006	0.73609	0.73524	0.64307	0.60229	0.87464
11/14/2020	0.83893	0.66354	0.82550	0.82550	0.01900	0.92626	0.36777	0.73249	0.72551	0.73204	0.63769	0.60080	0.86961
11/21/2020	0.84674	0.68477	0.85183	0.85183	0.02597	0.92919	0.39170	0.73197	0.73540	0.73900	0.64131	0.58701	0.86111
11/28/2020	0.84186	0.69583	0.85790	0.85790	0.03060	0.92847	0.41727	0.73501	0.74979	0.74804	0.64980	0.57644	0.85066
12/5/2020	0.84696	0.69824	0.86239	0.86239	0.03337	0.93090	0.42701	0.73856	0.74993	0.75157	0.64403	0.58264	0.84459
12/12/2020	0.85139	0.70241	0.86587	0.86587	0.04119	0.93096	0.40840	0.73606	0.75567	0.75266	0.62930	0.59229	0.84076
12/19/2020	0.85977	0.70670	0.86799	0.86799	0.05069	0.93034	0.38793	0.74969	0.76121	0.75460	0.62274	0.59737	0.84004
12/26/2020	0.86306	0.70747	0.86441	0.86441	0.05946	0.92813	0.37114	0.77123	0.76561	0.75540	0.63340	0.59917	0.83967

Table 1: Mask Compliance in 2020. IHME
